# Plastid genome evolution and phylogenomics with broad taxon sampling: insights into intrafamilial classification of Hamamelidaceae

**DOI:** 10.3389/fpls.2026.1926463

**Published:** 2026-08-26

**Authors:** Sadaf Habib, Yong Shi, Jie Zhang, Wyckliffe Omondi Omollo, Dennis Stevenson, Jinlong Li, Yangjun Lai, Chen Feng

**Affiliations:** 1 Jiangxi Provincial Key Laboratory of Ex Situ Plant Conservation and Utilization, Lushan Botanical Garden, Chinese Academy of Sciences, Jiujiang, China; 2 National Key Laboratory for Germplasm Innovation & Utilization of Horticultural Crops, Huazhong Agricultural University, Wuhan, China; 3 New York Botanical Garden, Bronx, NY, United States; 4 Shanxi Agricultural University, Taiyuan, China; 5 State Key Laboratory of Plant Diversity and Specialty Crops and Key Laboratory of Systematic and Evolutionary Botany, Institute of Botany, Chinese Academy of Sciences, Beijing, China; 6 China National Botanical Garden, Beijing, China

**Keywords:** complete sampling, evolution, Hamamelidaceae, phylogenetic, plastid genome

## Abstract

Hamamelidaceae, within the order Saxifragales, comprises 27 genera and approximately 120 species. The family has a pantropical and temperate distribution across the Americas, Asia, Africa, and Australia. Previous molecular investigations, constrained by limited taxon sampling and inadequate genetic markers, supported a five-subfamily classification system. However, these studies predominantly focused on Asian taxa, resulting in poor resolution of the evolutionary relationships among American, African, and Australian genera. To address these sampling gaps, we employed near-complete generic sampling (26 of 27 genera) to investigate plastome architecture, structural variation, and phylogenetic relationships. We newly sequenced and assembled 15 plastid genomes representing geographically and taxonomically underrepresented genera and analyzed them alongside 59 publicly available plastomes retrieved from GenBank. Plastid genomes exhibited conserved quadripartite architecture with sizes ranging from 158, 076 bp to 160, 814 bp, minimal structural variation, consistent GC content (37.7-38.2%), and identical gene order. Inverted repeat (IR) regions had limited size variation (26, 211-26, 429 bp). Simple sequence repeat (SSR) distribution (2, 219 loci) showed no clear correlation with the genus-level phylogenetic relationships. We identified ten hypervariable regions, including coding sequences (*accD, ycf1, clpP, ndhF*, and *rpl22*) and intergenic spacers (*rpl33-rps18*, the *trnG-UCC* intron, *trnH-GUG-psbA, accD-psaI*, and *petA-psbJ*), as promising candidate regions for future applications in species delimitation and phylogenetic studies. Phylogenetic analyses revealed largely congruent topologies across datasets and methods, providing improved resolution and strong support for most subfamilial and tribal relationships compared with previous studies. This study highlights the utility of plastid genome data for resolving deep-level phylogenetic relationships within Hamamelidaceae. The genome architecture reflects the high conservation of plastid genomes, while the identified mutation hotspots represent potential resources for future taxonomic and phylogenetic studies. Our results support the existing subfamily classification while improving geographical coverage and generic representation, providing a robust framework for future taxonomic and evolutionary studies of this globally distributed and taxonomically complex family.

## 1 Introduction

Hamamelidaceae is a diverse plant family consisting of 27 extant genera and approximately 120 species (POWO, 2026). Its members exhibit a wide geographic distribution, occurring in both temperate and tropical regions across Eastern Asia, North and Central America, Africa, Australia, and the Pacific Islands (Endress, 1993; Xiang et al., 2019). Several genera hold significant economic and cultural value. First, species within genera such as *Exbucklandia*, *Parrotiopsis, Mytilaria*, and *Trichocladus* provide valuable timber used in construction and furniture production (Meholic, 2019). Second, medicinal applications are prominent; notably, *Hamamelis* (witch hazel), *Fortunearia*, *Parrotiopsis*, and *Corylopsis* possess therapeutic properties utilized in traditional medicine (Kim et al., 2013; Ali et al., 2018; Meholic, 2019). Furthermore, genera like *Fothergilla*, *Rhodoleia*, and *Corylopsis* are highly valued for their ornamental qualities, making them popular subjects in horticulture and landscape design (Endress, 1993; Meholic, 2019).

The taxonomic and phylogenetic relationships within Hamamelidaceae have been the subject of sustained research interest for decades. Historically, taxonomic studies relied primarily on comparative morphology and anatomy, particularly leaf and floral characters, to develop the infrafamilial classification. Early systems, exemplified by the pioneering work of Harms (1930), divided the family into five subfamilies (i.e., Bucklandioideae (Exbucklandioideae), Disanthoideae, Hamamelidoideae, Liquidambaroideae (Altingioideae) and Rhodoleiodeae), based principally on characters such as floral symmetry, stamen number, and capsule dehiscence patterns. Harms further subdivided Hamamelidoideae into five tribes: Corylopsideae, Eustigmateae, Distylieae, Fothergilleae, and Hamamelideae. Chang (1973) erected a new subfamily Mytilarioideae for *Mytilaria* and *Chunia*. Subsequent research Endress (1989a, 1989b) refined this framework, incorporating additional morphological characters such as trichome structure and pollen morphology to better delineate genera and tribes, and subdivide Hamamelideae into three subtribes: Hamamelidinae, Dicoryphinae, and Loropetalinae. Nevertheless, morphology-based classifications faced persistent challenges because of the widespread occurrence of homoplasy in both vegetative and reproductive characters, frequently resulting in uncertain generic boundaries and conflicting tribal circumscriptions.

Molecular phylogenetic studies have revolutionized our understanding of relationships within Hamamelidaceae. Initially, investigations utilizing limited sequence variation, specifically from markers such as ITS, *mat*K, *rbc*L, and *trn* regions, began to reveal underlying phylogenetic conflicts. Subsequent analyses incorporating broader datasets, including both combined plastid (e.g., *atpB, matK, rbcL, atpB‐rbcL, trnH-GUG*‐*psbA, trnL‐trnF*) and nuclear (e.g., ITS) genes consistently demonstrated widespread non-monophyly among morphologically defined genera and tribes. This evidence precipitated major taxonomic reclassifications (Shi et al., 1998; Li et al., 1999a, 1999b). Notably, Li and Bogle (2001) revised the previous classification systems and presented a new suprageneric classification of Hamamelidoideae, based on combined molecular and morphological data. They divided the sub family into six tribes: Corylopsideae, Loropetaleae, Hamamelideae, Fothergilleae, Eustigmateae, and Dicorypheae. Subfamily Liquidambaroideae (Altingioideae) has subsequently been elevated to the rank of a separate family, Altingiaceae (APG, 1998; Magallón et al., 1999; Ickert-Bond and Wen, 2013). While these phylogenetic approaches have significantly clarified relationships, persistent gaps remain, particularly within under-sampled lineages. Consequently, expanded taxonomic sampling is essential to align nomenclature more closely with the emerging molecular topologies.

Next-generation sequencing (NGS) technologies have unlocked unprecedented opportunities for resolving long-standing phylogenetic and systematic questions by enabling the generation of genomic-scale datasets (Metzker, 2010). Plastid genomes, while generally conserved in structure and gene content, exhibit taxon-specific variations such as gene loss, inverted repeat (IR) expansions, structural rearrangements, and inversions (Gitzendanner et al., 2018a; Li et al., 2019; Ruhfel et al., 2014). These genomic dynamics render plastid genomes powerful tools for elucidating evolutionary relationships, population genetics, and diversification patterns (Gitzendanner et al., 2018b; Wen et al., 2021; Li et al., 2021; Yang et al., 2022; Liu et al., 2023). Within Hamamelidaceae, numerous plastid genomes have been sequenced to investigate plastid evolution and phylogenomic relationships at the genus and subfamily levels (Dong et al., 2021; Wang et al., 2022; Kim et al., 2024; Liu et al., 2025). However, current sampling efforts remain disproportionately focused on taxa from tropical Asia, creating significant gaps in representation from biogeographically critical regions such as North and Central America, Africa, and Australia. Given its morphological diversity and global distribution, Hamamelidaceae serves as an ideal model system for addressing taxonomic, phylogenetic, and biogeographic complexities. To advance this potential and resolve persistent evolutionary uncertainties, expanding both genomic datasets and taxon sampling, particularly from underrepresented regions, is essential for constructing robust phylogenetic frameworks within the family.

To address these limitations, this study employs an expanded genus-level sampling strategy by incorporating representatives from previously underrepresented lineages. By assembling and analyzing multi-locus plastid genome datasets spanning the family’s full taxonomic and geographic diversity, we aim to: (1) reconstruct a highly resolved backbone phylogeny to clarify deep evolutionary relationships and test current tribal classifications; (2) elucidate structural evolution within the plastid genome (including inversions, inverted repeat [IR] boundary dynamics, and gene loss); and (3) provide essential genomic resources to resolve persistent taxonomic uncertainties, thereby establishing a stable phylogenetic foundation for this ecologically and evolutionarily significant angiosperm family.

## 2 Material and methods

### 2.1 Plant sampling and genomic data acquisition

To improve phylogenetic sampling of Hamamelidaceae, particularly for geographically and taxonomically underrepresented genera, we newly sequenced and assembled 15 plastid genomes representing taxa from the Americas (*Molinadendron, Matudaea, Hamamelis, Fothergilla*), Africa (*Trichocladus*), Australia (*Neostrearia, Noahdendron, Ostrearia*), and Asia (*Maingaya Corylopsis, Parrotia*, and *Rhodoleia*). The corresponding specimens originated from botanical gardens or collaborators and were supported by herbarium vouchers and the necessary collection permits (**Table 1**). These newly generated plastomes were combined with 59 publicly available plastid genomes retrieved from GenBank (**Supplementary Table 1**), resulting in a dataset of 74 plastid genomes. The dataset included 69 in-group species representing 26 of the 27 recognized genera of Hamamelidaceae (excluding *Embolanthera*), and five outgroup species from allied families Altingiaceae, Cercidiphyllaceae and Daphniphyllaceae.

**Table 1:** Basic characteristics of the 15 plastid genomes newly assembled belonging to the family Hamamelidaceae.

No.	Species	Herbarium accession	Collection	NCBI accession	Total length (bp)	LSC (bp)	SSC (bp)	IR (bp)	GC (%)
1	*Corylopsis glabrescens*	Ratcliff & Stevenson 2024D (NY)	Living Collections: 465/93*B at New York Botanical Garden	PX119977	159, 434	88, 173	18703	26279	38
2	*Fothergilla gardenii*	Ratcliff & Stevenson 2024A (NY)	Living Collections: 939/98B, New York Botanical Garden	PX119978	159, 641	88, 264	18775	26302	38
3	*Hamamelis ovalis*	Ratcliff & Stevenson 2024E (NY)	Living Collections: 599/2009*A, New York Botanical Garden	PX119979	159, 732	88, 301	18763	26334	38
4	*Hamamelis vernalis*	Ratcliff & Stevenson 2024C (NY)	Living Collections: 621/2010A, New York Botanical Garden	PX119980	159, 729	88, 286	18761	26341	38
5	*Hamamelis virginiana*	Ratcliff & Stevenson 2024B (NY)	Living Collections: 941/98A, New York Botanical Garden	PX119981	159, 732	88, 391	18763	26289	38
6	*Maingaya malayana*	Y.J.Lai s.n. (PE)	Living Collections: China National Botanical Garden, Beijing, China.	PX119982	159, 396	87, 995	18849	26276	38.1
7	*Matudaea trinervia*	BG Chagala 491 (NY)	Pueblo, Mexico	PX119983	159, 236	87, 895	18771	26285	38.2
8	*Molinadendron hondurense*	K5305 (K)	Honduras	PX119984	159, 115	87, 935	18718	26231	38.2
9	*Neostrearia fleckeri*	QRS126728.1 (CNS)	Queensland, Australia	PX119989	158, 885	87, 437	18856	26296	38.2
10	*Noahdendron nicholasii*	CNS153476.1 (CNS)	Queensland, Australia	PX119990	158, 707	87, 348	18867	26246	38.1
11	*Ostrearia australiana*	CNS153845.1 (CNS)	Queensland, Australia	PX119991	158, 584	87, 437	18589	26279	38.1
12	*Parrotia persica*	Zhang S.Z. 20240728 (SZG)	Living Collections: Royal Botanical Garden of Madrid, Spain	PX119985	159, 166	87, 839	18811	26258	38
13	*Rhodoleia forrestii*	E19406 (E)	Yunnan, China	PX119986	158, 856	87, 986	18140	26365	37.7
14	*Trichocladus crinitus*	E5641 (E)	Natal, South Africa	PX119987	158, 733	87, 162	18949	26311	38
15	*Trichocladus grandiflorus*	E12645 (E)	Transvaal, South Africa	PX119988	158, 445	86, 978	18831	26318	38.1

CNS, Australian National Herbarium, Canberra, Australia; E, Royal Botanic Garden, Edinburgh, U.K; K, Royal Botanic Gardens, Kew, U.K; NY, The New York Botanical Garden, New York, U.S.A; PE, Institute of Botany, Chinese Academy of Sciences, Beijing, China; SZG, Fairy Lake Botanical Garden, Shenzhen, China.

For newly sequenced taxa, we extracted total genomic DNA from herbarium specimens and fresh leaves using a modified cetyl trimethyl ammonium bromide (CTAB) protocol (Doyle and Doyle, 1987) and purified it with the DNeasy Plant Mini Kit (Qiagen). We verified DNA integrity through 1% agarose gel electrophoresis and quantified concentration/purity using Qubit Fluorometric Quantitation (Thermo Fisher Scientific). After fragmenting qualified DNA samples to ~500 bp, we constructed paired-end libraries with the Illumina TruSeq Nano DNA HT Kit and sequenced them on an Illumina HiSeq 2500 platform (Novogene) with 150-bp reads generating 4–6 Gb raw data per sample.

We preprocessed raw reads with Trimmomatic v0.39 (Bolger et al., 2014) to remove adapters, low-quality bases (LEADING:3, TRAILING:3, SLIDINGWINDOW:4:15), and discard reads shorter than 36bp (MINLEN:36). Using *rbcL* from *Corylopsis microcarpa* (NC_061208) as a seed reference, we *de novo* assembled plastid genomes in NOVOPlasty v2.7.2 (Dierckxsens et al., 2017). We annotated assemblies in GeSeq v1.0 (Tillich et al., 2017), manually verified them in Geneious Prime v2025 (https://www.geneious.com) against reference plastomes (*Corylopsis microcarpa* NC_061208), and visualized genomes with OGDRAW v1.3.1 (Greiner et al., 2019). Newly generated plastid genomes were submitted to NCBI database (accession numbers: PX119977-PX119991; **Table 1**).

### 2.2 Comparative genomics and structural analyses

We characterized structural variability across Hamamelidaceae by comparing 28 representative accessions spanning major lineages and genera, determining junction sites of plastid genome regions, large single copy (LSC), small single copy (SSC), and inverted repeats (IRA/IRB), using IRplus (Díez Menéndez et al., 2023) to quantify IR boundary expansions/contractions. We detected DNA rearrangements via Mauve Alignment (Darling et al., 2010) in Geneious Prime v.2025 and identified simple sequence repeats (SSRs) with the MISA Perl script (Beier et al., 2017), applying thresholds of ≥ 10 repeat units for mononucleotides, ≥ 5 for dinucleotides, ≥ 4 for trinucleotides, and ≥ 3 for tetra-, penta-, and hexanucleotides. To assess sequence divergence in coding regions, non-coding regions, and introns, we calculated nucleotide diversity (π) using DnaSP v6.12.03 (Rozas et al., 2017), excluding alignments shorter than 200 bp due to insufficient phylogenetic information from limited sequence variation.

### 2.3 Phylogenetic reconstruction

To reconstruct the evolutionary history of Hamamelidaceae, we generated three sequence matrices from whole plastid genomes for phylogenetic analyses: (1) protein-coding genes (PCG), (2) non-coding regions (NCG), and (3) whole plastid genomes excluding one inverted repeat (WP). One copy of inverted repeat (IR) region was removed from NCG and WP matrices prior to alignment to avoid overweighting duplicated phylogenetic signals. The resulting sequences were then aligned using MAFFT v7.463 (Katoh and Standley, 2013) with the AUTO strategy. For the PCG matrix, protein coding genes were extracted individually from each plastome. For genes duplicated in IR regions, only a single copy was retained for alignment and concatenation using Geneious Prime v.2025.

We implemented both concatenation and coalescent approaches for phylogenetic inference. For concatenated analyses, the optimal partitioning scheme for the PCG dataset was first determined using PartitionFinder v2.1.1 (Lanfear et al., 2016). Maximum likelihood (ML) trees for the PCG, NCG, and WP matrices were then inferred using IQ-TREE v2.4.0 (Minh et al., 2020) with 1, 000 ultrafast bootstrap replicates. We simultaneously determined optimal substitution models via ModelFinder (Kalyaanamoorthy et al., 2017) under the Bayesian Information Criterion (BIC). For coalescent analyses, gene trees for each of the 81 PCGs across the 74 taxa were inferred using IQ-TREE, with the best-fit substitution model for each gene selected using ModelFinder under the BIC. The resulting gene trees were subsequently integrated into wASTRAL v1.1 (Zhang and Mirarab, 2022) under hybrid weighting (wASTRAL-h) to estimate the species tree, with node supports assessed using local posterior probabilities.

## 3 Results

### 3.1 Chloroplast genome features and comparative structure

**Figure 1:**
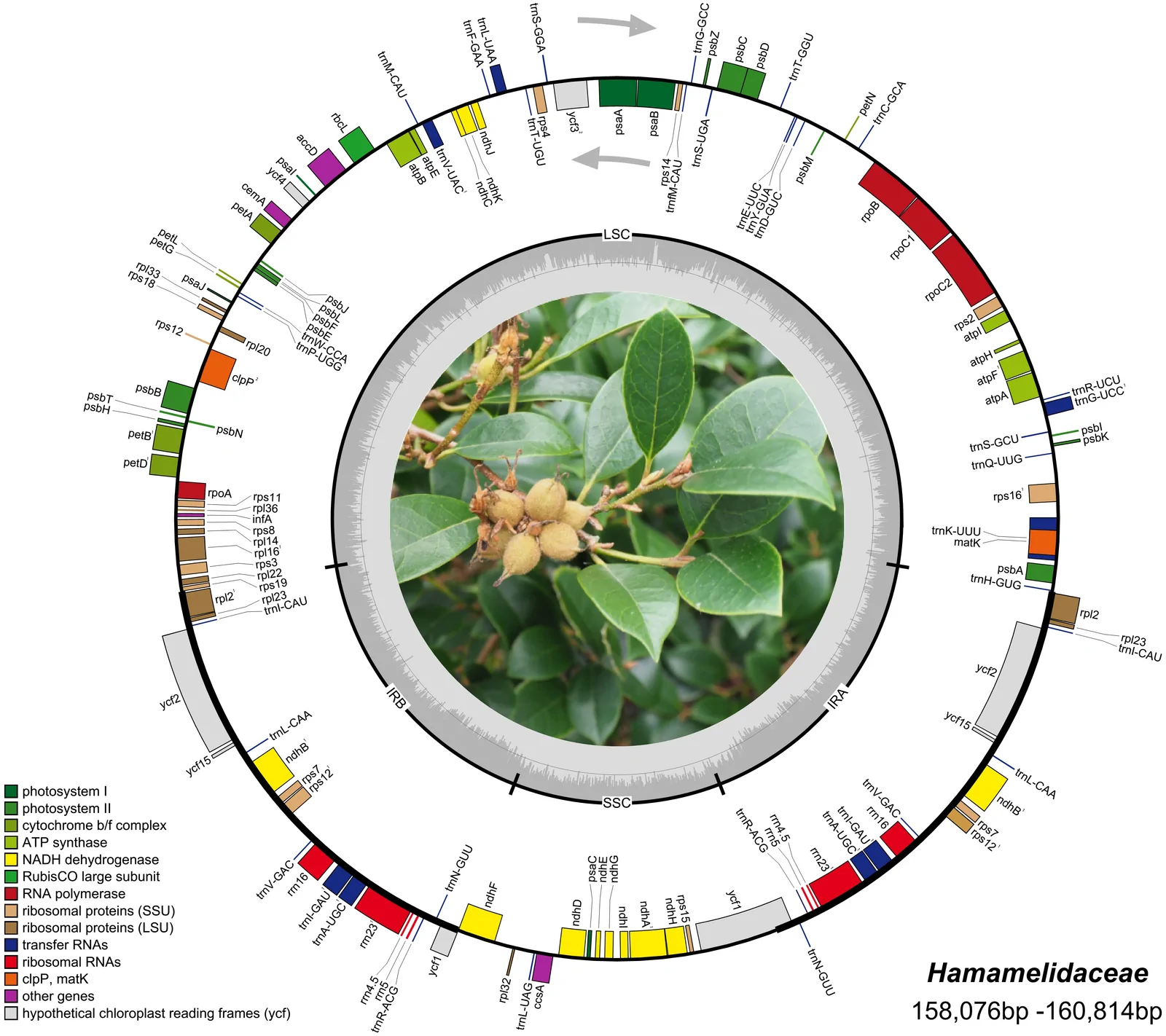
Whole chloroplast genome map of Hamamelidaceae species. Genes shown outside of outer circle are transcribed clockwise, while those insides are transcribed counterclockwise. The genes are color-coded according to the different functional groups they belong. The light and dark gray area of the inner circle denotes the GC and AT content of plastid genome, respectively.

The complete chloroplast genomes of Hamamelidaceae species ranged in size from 158, 076 bp (*Disanthus cercidifolius* subsp. *longipes*) to 160, 814 bp (*Exbucklandia tonkinensis*) (**Figure 1**). These genomes exhibited a typical quadripartite structure that consists of two inverted repeat regions (IRa and IRb; 26, 211–26, 429 bp) separated by a large single-copy region (LSC; 86, 918–89, 060 bp) and a small single-copy region (SSC; 18, 127–18, 931 bp) (**Figure 1**, **Table 1**). The Hamamelidaceae plastid genomes contained 115 unique genes, representing 81 unique protein-coding genes (PCGs), 30 transfer RNA (tRNA) genes, and four ribosomal RNA (rRNA) genes. Among these, 17 genes were duplicated in the IR regions, comprising six PCGs (*ndhB*, *rpl2*, *rpl23*, *rps7*, *rps12*, *ycf2*), seven tRNAs (*trnA-UGC*, *trnI-CAU*, *trnI-GAU*, *trnL-CAA*, *trnN-GUU*, *trnR-ACG*, *trnV-GAC*), and four rRNAs (*rrn16*, *rrn23*, *rrn4.5*, *rrn5*). Accounting for these IR duplications, total number of genes copies per plastid genome is 132. Nine PCGs (*atpF*, *ndhA*, *ndhB*, *petB*, *petD*, *rpl2*, *rpl16*, *rpoC1*, *rps16*) and six tRNAs (*trnA-UGC*, *trnG-UCC, trnI-GAU*, *trnK-UUU*, *trnL-UAA*, *trnV-UAC*) contained a single intron, while three PCGs (*clpP*, *rps12, ycf3*) possessed two introns. Notable exceptions included the absence of *ycf68* in *Noahdendron nicholasii* and the pseudogenization of *ycf15* in *Chunia bucklandioides* and *psbL* in *Trichocladus crinitus*. The GC content across the examined plastid genomes was relatively conserved, ranging from 37.7% (*Rhodoleia forrestii*) to 38.2% (observed in three species: *Matudaea trinervia, Sinowilsonia henryi*, and *Molinadendron hondurense*).

Mauve alignment analysis across the 28 investigated taxa demonstrated good collinearity with no evidence of gene rearrangement or inversion (**Supplementary Figure 1**). However, variations were evident at the inverted repeat/single copy (IR/SC) boundary regions (**Supplementary Figure 2**). The primary factor accounting for differences in plastid genome size among species was the expansion and contraction of these LSC and SSC regions. Comparative analysis of IR boundary positions revealed non-identical placements of border genes among the examined plastid genomes (**Supplementary Figure 2**). Specifically, at the large single copy/inverted repeat B (LSC/IRb border or JLB) junction, the *rps19* gene was either positioned entirely within the LSC region or spanned the JLB boundary. Near the IRb/small single copy (IRb/SSC or JSB) junction, two genes of highly variable length (*ψycf1* and *ndhF*) were located. These genes exhibited slight overlap, ranging from 3 bp in *Loropetalum chinense* to 101 bp in *Exbucklandia tonkinensis* or no overlap, as observed in *Rhodoleia forrestii*. At the SSC/IRa (JSA) junction, the *ψycf1* gene was distributed almost equally on both sides of the boundary. The IRa/LSC (JLA) junction consistently lay between the *rpl2* and *trnH-GUG* genes. While the r*pl2* gene was primarily located within the IR region adjacent to JLA, a small segment (3–6 bp) extended into the LSC region in *Noahdendron nicholasii* and *Matudaea trinervia*. The *trnH-GUG* gene was invariably positioned within the LSC region, located 0–64 bp away from the JLA junction (**Supplementary Figure 2**). These variations in boundary positions likely influence genome structure and gene content.

**Figure 2:**
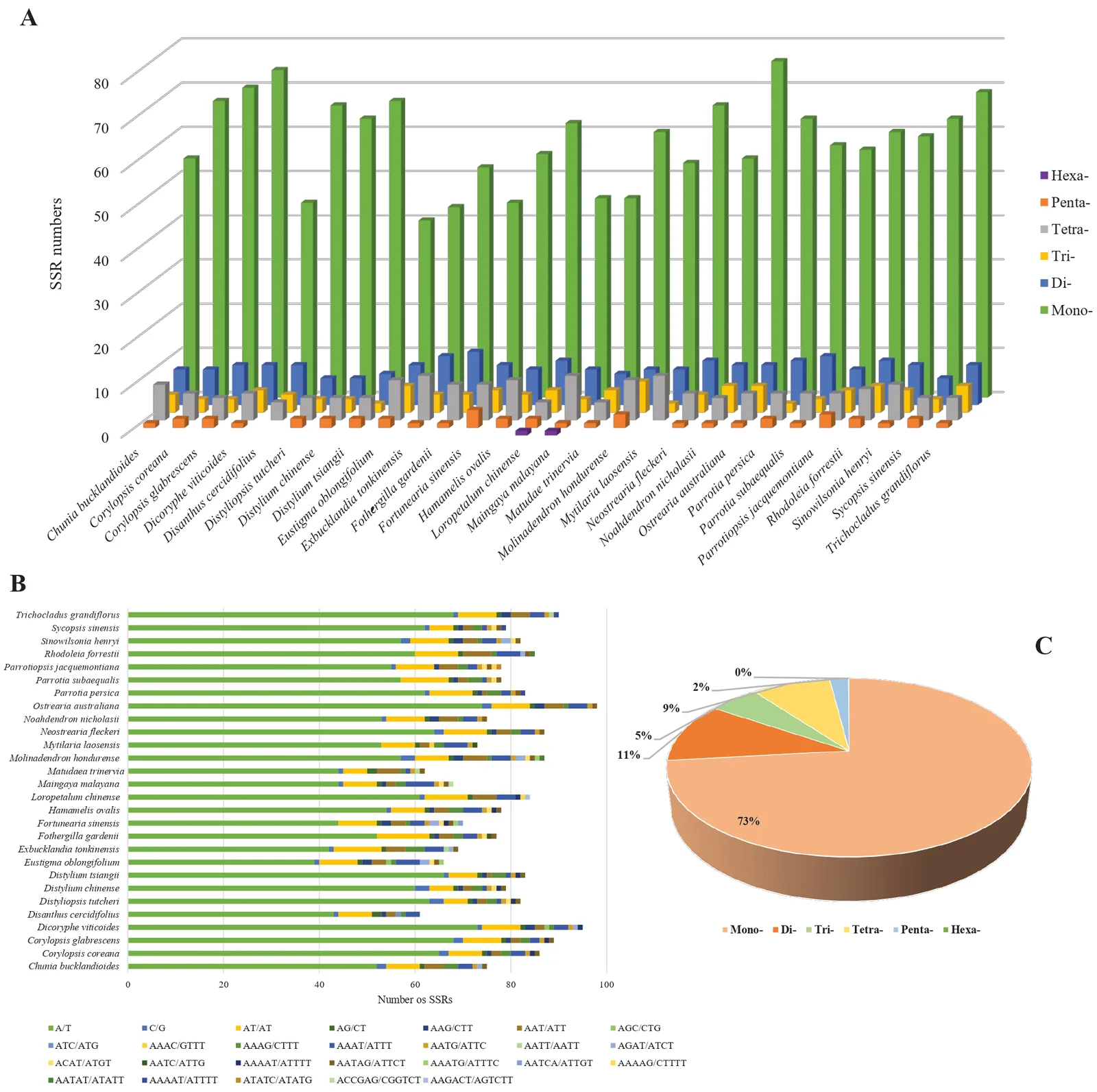
Analysis of simple sequence repeats (SSRs) in the twenty-eight plastid genomes representing all the genera included in this study. **(A)** Number of different types of SSR detected in the genomes; **(B)** Number of different SSR motifs in the genomes; **(C)** Statistics of the overall proportion of different types of SSR.

An analysis of simple sequence repeats (SSRs) identified a total of 2, 219 SSR loci across the 28 representative Hamamelidaceae plastid genomes (**Figure 2**; **Supplementary Table 2**). Mononucleotide SSRs were the most abundant (1, 627 loci, 73.3%), whereas hexanucleotide SSRs were the least frequent (2 loci, <1%). The highest number of mononucleotide SSRs was found in *Ostrearia australiana* (76 loci), followed by *Dicoryphe viticoides* (74 loci) and *Corylopsis glabrescens* (70 loci), whereas *Exbucklandia tonkinensis* had the fewest (43 loci) (**Supplementary Table 2**). Total SSR counts ranged from 61 in *Disanthus cercidifolius* to 98 in *Ostrearia australiana*, with a median of 79 SSRs, represented by *Distylium chinense* and *Sycopsis sinensis* (**Supplementary Table 2**). Among SSR types, mononucleotide A/T motifs (1, 592) were the most abundant, followed by dinucleotide AT/TA motifs (214 loci) (**Supplementary Table 3**).

### 3.2 Divergence hotspots

**Figure 3:**
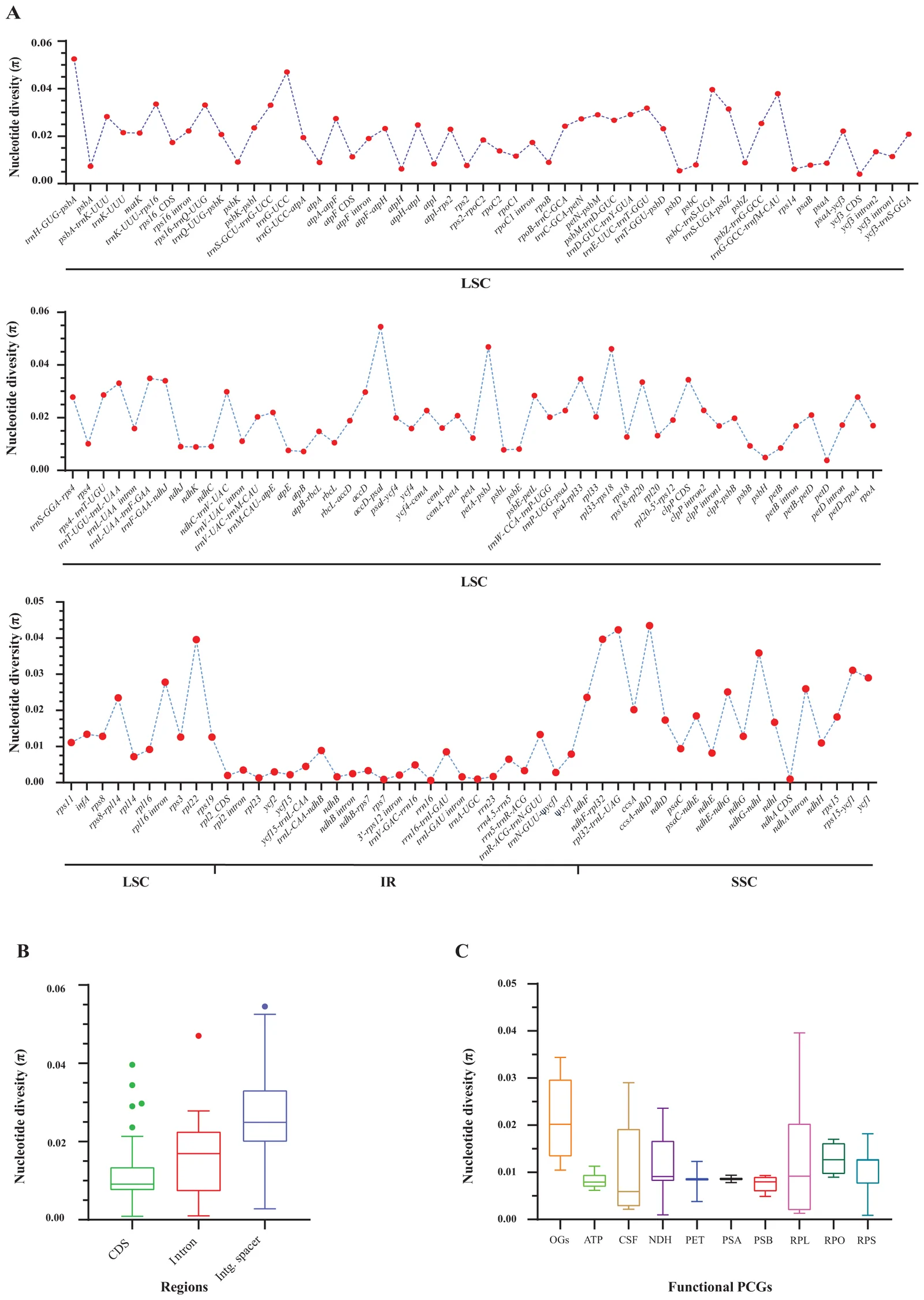
Comparison of the nucleotide diversity (π) values in twenty-eight chloroplast genomes of Hamamelidaceae. **(A)** The nucleotide diversity (π) in chloroplast regions (LSC/IR/SSC). **(B)** The nucleotide diversity (π) in protein coding genes (PCGs), introns and Non-coding intergenic regions. **(C)** The nucleotide diversity (π) of different functional groups. Detailed information of nucleotide diversity among plastid genomes of investigated taxa is provided in **Supplementary Table 4**.

To identify highly variable regions, nucleotide diversity (π) was calculated for 157 aligned loci > 200 bp, including coding sequences and non-coding regions (introns and intergenic spacers). The π values ranged from 0.0006 in *rrn16* to 0.05445 in *accD-psaI*, with most loci exhibiting intermediate diversity (π = 0.01–0.02) among Hamamelidaceae species (**Figure 3**; **Supplementary Table 4**). Comparative analysis revealed consistently lower nucleotide diversity in the inverted repeat (IR) regions (median π = 0.0027) than in the large single-copy (LSC; median π = 0.0193) and small single-copy (SSC; median π = 0.0185) regions (**Figure 3A**; **Supplementary Table 4**). Non-coding intergenic spacers exhibited higher nucleotide diversity than coding regions (**Figure 3B**, **Supplementary Table 4**). Functional classification of coding regions further showed the “other genes” (OG) category had the highest median π, while genes associated with ATP synthase (ATP), cytochrome b/f complex (PET) and photosystems I (PSA) and II (PSB) displayed comparatively lower median values, consistent with their evolutionary conservation (**Figure 3C**; **Supplementary Table 4**). This heterogeneity in nucleotide diversity reflects differential functional constraints across the plastid genomes.

### 3.3 Phylogenetic analyses

Phylogenetic relationships within Hamamelidaceae were inferred using three datasets: whole plastid genomes (WP), non-coding regions (NCG), and protein-coding genes (PCGs) from a dataset of 74 taxa, including 69 Hamamelidaceae species and five outgroup species. Concatenated alignments spanned 172, 259 bp for WP (15, 624 distinct sites and 13, 455 parsimony-informative sites), 74, 514 bp for NCG (12, 896 distinct sites and 8, 294 parsimony-informative sites), and 70, 329 bp for PCGs (7, 571 distinct sites and 4, 591 parsimony-informative sites). PartitionFinder identified the optimal partitioning scheme for the PCG dataset, resulting in three partitions corresponding to the first, second, and third codon positions. ModelFinder selected best-fit partitioning schemes and substitution models based on the Bayesian Information Criterion (BIC): K3Pu+F+R4 for WP and K3Pu+F+R3 for NCG, while codon-partitioned PCGs used TVM+F+R2 for positions 1 & 2 and TVM+F+R3 for position 3 (**Supplementary Table 5**).

**Figure 4:**
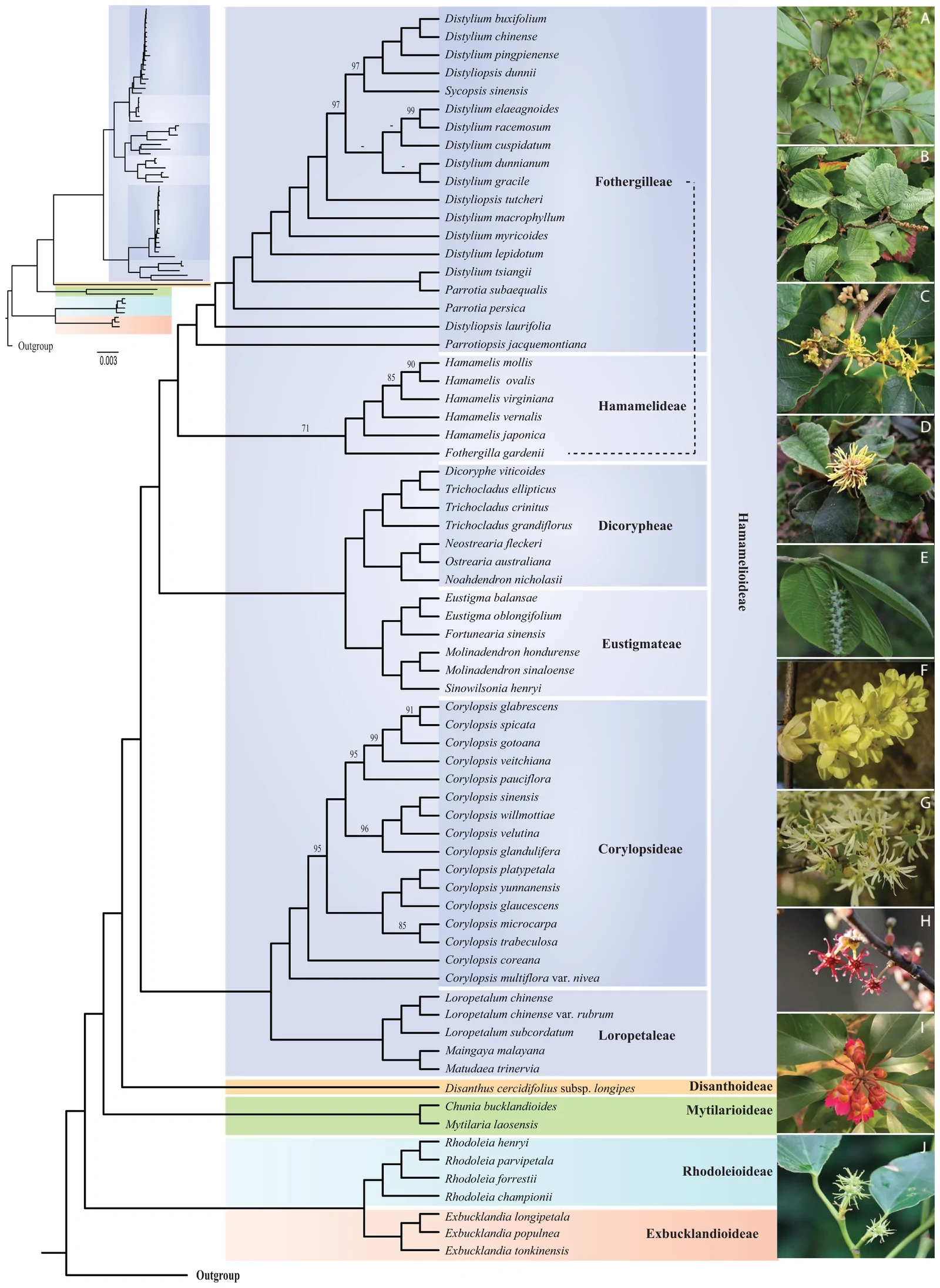
Maximum likelihood (ML) phylogenetic tree of 69 Hamamelidaceae species inferred from the whole plastid genome (WP) dataset. Five outgroup species were included in the phylogenetic analysis to root the tree but are not shown. Values along branches represent ML bootstrap percentages (only BS < 100% are shown). The dotted line shows the conflicting position of *Fothergilla gardenii* among two tribes based on different datasets. Photographs on the right side of the phylogenetic tree show representative taxa from the major groups of Hamamelidaceae proposed in this study. Subfamily Hamamelidoideae: **(A)** *Sycopsis sinensis* (tribe Fothergilleae), **(B)** *Fothergilla major* (tribe Fothergilleae), **(C)** *Hamamelis virginiana* (tribe Hamamelideae), **(D)** *Trichocladus crinitus* (tribe Dicorypheae), **(E)** *Sinowilsonia henryi* (tribe Eustigmateae), **(F)** *Corylopsis sinensis* (tribe Corylopsideae), and **(G)** *Loropetalum chinense* (tribe Loropetaleae); subfamily Disanthoideae: **(H)** *Disanthus cercidifolius* subsp. *longipes*; subfamily Rhodoleioideae: **(I)** *Rhodoleia> championii*; subfamily Exbucklandioideae: **(J)** *Exbucklandia populnea*. Photographs by Yangjun Lai **(A–G, I, J)** and Feng Chen **(H)**.

Maximum likelihood (ML) phylogenies from concatenated datasets (WP, NCG, PCGs) showed higher resolution than coalescent analysis (PCGs-coal); thus, effectively resolving sister-group relationships within major clades (**Figure 4**; **Supplementary Figure 3**). Topologies were largely congruent across datasets, differing principally in bootstrap support for inter-generic and inter-specific nodes. However, all datasets yielded weak statistical support for the placement of *Fothergilla*, *Sycopsis sinensis, Corylopsis coreana*, and *C. pauciflora*. Most genera were monophyletic, however, *Distyliopsis* species and *Sycopsis sinensis* were nested within *Distylium, Parrotia* was paraphyletic, and *Trichocladus* was paraphyletic with *Dicoryphe viticoides* sister to *T. ellipticum* (**Figure 4**; **Supplementary Figure 3**). Despite these localized uncertainties, analyses consistently recovered a well-resolved backbone comprised of five major clades (**Figure 4**; **Supplementary Figure 3**); thus, highlighting the efficacy of concatenated plastid genomes for deep phylogeny reconstruction while revealing persistent challenges at shallow evolutionary depths.

## 4 Discussion

### 4.1 Comparison of plastid genomes features

Plastid genome size in angiosperms exhibits substantial variation, ranging from 107 kb in Pinaceae to 218 kb in Geraniaceae (Zhang et al., 2012; Daniell et al., 2016). Within this range, the inverted repeat (IR) region is typically conserved between 20 and 30 kb (Zhang et al., 2012; Daniell et al., 2016). Analyses in this study reveal that plastid genome sizes across Hamamelidaceae cluster near the angiosperm median. Notably, divergence exists within the family: *Exbucklandia* possesses relatively large genomes (160, 814 bp), whereas *Disanthus cercidifolius* subsp. *longipes* has evolved a smaller genome (158, 076 bp). This size variation within Hamamelidaceae shows no consistent correlation with phylogenetic divergence or branching order. For instance, the sister genera *Exbucklandia* and *Rhodoleia* exhibit divergent plastid genome sizes of approximately 160, 814 bp and 158, 856 bp, respectively. Paradoxically, despite the family’s phylogenetic diversity, plastid gene content remains highly conserved, with uniform numbers of tRNA and protein-coding genes across taxa. In particular, the protein-coding regions (PCGs) exhibit the highest degree of sequence conservation.

While IR expansion/contraction is a major driver of plastid genome size variation in angiosperms (Zhu et al., 2016), our analyses indicate minimal variation in IR length among Hamamelidaceae plastid genomes. Only *Exbucklandia* and *Mytilaria* exhibited slight IR expansions, suggesting limited structural divergence compared to other angiosperm lineages (**Table 1**; **Supplementary Figure 2**). This IR conservation is critical for maintaining plastid genome structural stability (Maréchal and Brisson, 2010). Consequently, size variation in this family is primarily attributed to differences in the lengths of the large single-copy (LSC) and small single-copy (SSC) regions. This aligns with the observation that point mutations accumulate more slowly in IR regions than in single-copy (SC) regions (Zhu et al., 2016). Specifically, *Exbucklandia* and *Dicoryphe* display expanded SSC regions, while *Mytilaria* and *Exbucklandia* possess the largest LSC regions (**Table 1**; **Supplementary Figure 2**). Notably, the larger plastid genome of *Exbucklandia* is associated with expansions in both the LSC and SSC regions. Such SC region-driven size variation is consistent with patterns documented in other diverse angiosperm lineages (Bayly et al., 2013; Sun et al., 2016; Chen et al., 2021).

Simple sequence repeats (SSRs), short tandem repeats (1–6 bp) pervasive in coding and intergenic regions, contribute to genomic stability and serve as powerful tools for assessing genetic diversity, population structure, phylogenetic relationships, and conservation strategies due to their high mutational variability and genome-wide distribution (Ellegren, 2004; Liu et al., 2020). Across the 28 Hamamelidaceae plastid genomes examined, we identified 2, 219 SSRs. Mononucleotide repeats were the most abundant (73%), followed by dinucleotide (11%), trinucleotide (9%), tetranucleotide (5%), pentanucleotide (2%), and hexanucleotide (0.1%) repeats. Consistent with previously reported SSR patterns in *Corylopsis*, *Distylium*, and other Asian genera (Dong et al., 2021; Kim et al., 2024; Liu et al., 2025), a striking predominance of A/T-rich motifs was observed, reinforcing the overall AT-biased composition of Hamamelidaceae plastid genomes. SSR distribution showed inconsistent variation across closely related genera, with no clear lineage-specific pattern evident. Notably, a tetranucleotide repeat motif (AAAT/ATTT) was identified in high abundance (83 loci), highlighting its potential as a polymorphic marker for population genetic and phylogeographic studies within the family.

### 4.2 Hypervariable regions at genus level

Comparative analysis of plastid genome nucleotide diversity (π) is a robust method for identifying divergence hotspots that are critical for species delimitation and phylogenetic reconstruction across taxonomic scales (Ruhlman and Jansen, 2014; Li et al., 2021). Non-coding regions typically exhibit higher sequence variability than coding regions, enhancing their utility for distinguishing closely related taxa (Yang et al., 2022; Song et al., 2024). While universal plastid barcodes like *matK, rbcL*, and *trnH-GUG-psbA* are widely used in land plants (Kress and Erickson, 2007; CBOL Plant Working Group, 2009), lineage-specific studies within Hamamelidaceae have suggested alternative markers. For example, *trnH-GUG-psbA*, *psbJ-petA*, and *ycf1* have been proposed for subfamily-level studies based on analysis of 12 species (Liu et al., 2025), whereas *ndhE* and *rpl33-rps18* were suggested for *Corylopsis* (Kim et al., 2024), and *matK-trnK*, *ndhC-trnV*, *ycf1*, and *trnT-trnL* for *Distylium* (Dong et al., 2021).

Selecting effective hypervariable plastid regions as molecular markers requires careful consideration. High nucleotide diversity (π) is desirable, but very short sequences may lack sufficient informative sites despite high variability. Consequently, optimizing the balance between π and sequence length is critical to ensure the robust utility of these markers for resolving phylogenetic relationships or population genetic diversity. In our comparative analysis, we identified the five non-coding plastid regions with the highest nucleotide diversity (π) as *rpl33-rps18*, the *trnG-UCC* intron, *trnH-GUG-psbA*, *accD-psaI*, and *petA-psbJ*. Among coding regions, *rpl22*, *clpP*, *accD*, *ycf1*, and *ndhF* showed elevated variability (π > 0.02) relative to other plastid genes (**Table 2**). All identified high-π loci reside within the large single-copy (LSC) region, except *ycf1* and *ndhF*, which are positioned at the boundaries of the SSC and IR regions. Consistent with previous findings (Dong et al., 2021; Kim et al., 2024; Liu et al., 2025), conventional markers *rbcL* (π = 0.0105) and *matK* (π = 0.0213) demonstrated limited resolution at both intrafamilial and genus levels, in this study.

**Table 2:** Characteristics of 10 hypervariable regions within the family Hamamelidaceae.

Regions	Name	Total length (aligned)	Distinct sites	Parsimony-informative sites	Constant sites	Nucleotide diversity
Coding	*rpl22*	521	164 (31.5%)	99 (19%)	331 (63%)	0.0396
	*clpP*	598	133 (22.2%)	80 (13.4%)	500 (83.6%)	0.0344
	*accD*	1563	235 (15%)	140 (9%)	1301 (83.2%)	0.0297
	*ycf1*	5935	1118 (18%)	888 (15%)	4544 (76.6%)	0.0290
	*ndhF*	2250	472 (21%)	303 (13%)	1787 (79.4%)	0.0236
Non-coding	*rpl33-rps18*	215	77 (35.8%)	29 (13.5%)	153 (71.2%)	0.0461
	*trnG-UCC*	819	171 (20.9%)	58 (7.1%)	217 (26.0%)	0.0470
	*trnH-GUG-psbA*	467	178 (38%)	59 (12.6%)	336 (71.9%)	0.0525
	*accD-psaI*	850	286 (33.6%)	95 (11.2%)	672 (79.1%)	0.0545
	*petA-psbJ*	1157	330 (28.5%)	94 (8.1%)	854 (73.8%)	0.04686

Notably, five loci highlighted in our findings (*trnH-GUG-psbA, ycf1, rpl22, rpl33-rps18*, and *petA-psbJ*) correspond to divergence hotspots identified in prior Hamamelidaceae studies (Dong et al., 2021; Kim et al., 2024; Liu et al., 2025). Nevertheless, the utility of these regions as reliable DNA barcodes for species differentiation and resolving phylogenetic relationships within the family requires rigorous validation. Expanded intra- and interspecific sampling is essential to confirm their taxonomic reliability and robustness.

### 4.3 Plastid phylogeny in association with traditional classification of Hamamelidaceae

The phylogenetic framework proposed in this study reflects emerging consensus regarding subfamilial and tribal relationships within Hamamelidaceae and offers a robust evolutionary hypothesis grounded in comprehensive phylogenomic data. Our analyses yielded highly resolved trees with strong statistical support, even for nodes previously characterized with limited confidence or sparse molecular data in earlier studies (Li et al., 1999a, 1999b; Xiang et al., 2019). By contextualizing major clades within historical classification systems, we clarify intrafamilial and intergeneric relationships, thereby bridging traditional taxonomic frameworks with contemporary genomic insights. This approach not only validates key aspects of prior hypotheses but also advances the resolution of long-debated evolutionary relationships within the family.

Our analyses provide robust support for the monophyly of five subfamilies of Hamamelidaceae mentioned in previous classification systems (Harms, 1930; Endress, 1989b; Li et al., 1999a), namely Disanthoideae, Exbucklandioideae, Mytilarioideae, Rhodoleioideae, and Hamamelidoideae. This classification based primarily on distinctive floral characters unique to each subfamily is now supported by molecular data, making it even more robust. The earliest diverging clade is comprised of the sister subfamilies Exbucklandioideae and Rhodoleioideae. While both share capitate (head-like) inflorescences, Rhodoleioideae is distinguished by possessing 10–20 ovules per gynoecium (multiovulate), a feature absent in other subfamilies (Endress, 1989b). The subsequently diverging Mytilarioideae, containing the monotypic genera *Mytilaria* and *Chunia*, is characterized by spicate inflorescences and multilacunar nodal anatomy (Li, 1997; Bogle, 1990, 1991). Disanthoideae, the fourth diverging subfamily, is sister to Hamamelidoideae and is unique in having paired-flower inflorescences. This clade possesses a reduced stamen number (4–5 stamens) per flower that distinguishes it from the earlier diverging Rhodoleioideae + Exbucklandioideae and Mytilarioideae, with 10 or more stamens per flower (Endress, 1989b, 1993) and shows closer phylogenetic affinity to Hamamelidoideae (**Figure 4**; **Supplementary Figure 3**).

Hamamelidoideae representing the most species-rich and most recently diverged subfamily within Hamamelidaceae is characterized by a constant ovule number (one per carpel) that contrasts with variable more numerous ovule numbers in the other subfamilies (Endress, 1989a). Phylogenetic reconstruction supports its division into six tribes forming two well-supported monophyletic groups: one contains the sister tribes Loropetaleae and Corylopsideae, while the second consists of Eustigmateae sister to Dicorypheae, and Hamamelideae sister to Fothergilleae, largely aligning with Li and Bogle’s (2001) revised tribal classification of Hamamelidoideae. Tribe Loropetaleae is defined by distinctive adaxial anther connective protrusions and bisexual flowers (Endress, 1989a), and is comprised of *Loropetalum*, *Maingaya*, *Matudaea*, and *Embolanthera* (missing genus in current study). The monogeneric tribe Corylopsideae contains *Corylopsis* and *C. multiflora* var. *nivea* represents the earliest diverging lineage and the remaining taxa form two major clades with conflicting positions for species like *C. coreana, C. glabrescens, C. pauciflora*, and *C. glandulifera*. Morphologically, *Corylopsis* species share clawed orbicular petals, a seed caruncle, and polyembryony. Tribe Eustigmateae comprises *Eustigma* (sister to *Fortunearia*) and *Molinadendron* (sister to *Sinowilsonia*), characterized by linear stipules and dual prophylls per branch. Tribe Dicorypheae includes five Southern Hemisphere genera (*Dicoryphe, Neostrearia, Noahdendron, Ostrearia*, *Trichocladus*) sharing tetrasporangiate bithecate anthers with unique univalvate thecal dehiscence (Endress, 1989b). The phylogenetic relationships between the tribes Hamamelideae and Fothergilleae remain poorly resolved, primarily because of the conflicting placements of *Fothergilla* relative to these tribes (**Figure 4**; **Supplementary Figure 3**). Support for *Fothergilla* as sister to *Hamamelis* was weak in the WP (BS = 71), NCG (BS = 70), and PCGs-coal (PP = 0.45) analyses. In contrast, the placement of *Fothergilla* within Fothergilleae received stronger support in the PCGs-concat (BS = 87) analysis. Morphologically, *Hamamelis* shares semicraspedodromous leaf venation with Fothergilleae genera (*Parrotia*, *Parrotiopsis*), but differs significantly in floral structure. *Hamamelis* possesses strictly tetramerous flowers with distinct petals, develops precarpellary staminodial nectaries, and is bisexual (Mione and Bogle, 1990). In contrast, Fothergilleae flowers are apetalous and andromonoecious, and exhibit variable stamen numbers (Bogle, 1970). Within Fothergilleae, the genera *Distylium*, *Distyliopsis*, and *Parrotia* were distinctly non-monophyletic. Specifically, *Distyliopsis* and *Sycopsis* species were nested within *Distylium*, while *Parrotia* was paraphyletic with *P. subaequalis* as sister to *Distylium tsiangii* and *P. persica* sister to that clade. These findings support the circumscription of Fothergilleae *sensu* Endress (1989a) rather than the separate tribes Distylieae (*Distylium*, *Sinowilsonia*, *Sycopsis*) and Fothergilleae (*Fothergilla*, *Parrotia*, *Parrotiopsis*) as recognized in Harms’s classification (1930). Given the current dataset, the conflicting placement of *Fothergilla* and the para- or polyphyletic relationships within Fothergilleae remain unresolved. To advance phylogenetic resolution and establish a stable, unified nomenclature for Fothergilleae, an expanded taxonomic sampling that targets non-monophyletic genera, coupled with more molecular data acquisition, is imperative. Furthermore, the integration of nuclear genomic data will be critical for resolving phylogenetic uncertainties within the Corylopsideae and Hamamelideae/Fothergilleae lineages as well as evaluating underlying biological processes such as hybridization and incomplete lineage sorting. This integrated approach will clarify the evolutionary relationships and address lingering taxonomic ambiguities within these complex groups.

## 5 Conclusion

This study sequenced and assembled complete plastid genomes from fifteen Hamamelidaceae taxa with a focus on underrepresented genera. Integrating these data with existing plastid genomes enabled comprehensive comparative analyses across nearly all genera within the family. These analyses revealed highly conserved genome structures, consistent gene content, and uniform GC content among the 28 plastid genomes examined. Genomic size variation was primarily attributed to differences in the LSC and SSC regions, while the IR regions exhibited limited variation. We identified ten mutation hotspots exhibiting elevated variability, encompassing both coding regions (*accD, ycf1, clpP, ndhF, rpl22*) and noncoding intergenic spacers (*rpl33-rps18*, the *trnG-UCC* intron, *trnH-GUG-psbA, accD-psaI*, and *petA-psbJ*). These loci represent promising molecular resources for future studies of species delimitation and resolving phylogenetic relationships within this taxonomically complex lineage. Plastid phylogenomic analyses based on the assembled genome sequences, improved phylogenetic support and resolution. The inferred species relationships generally aligned with established subfamily and tribal classifications. However, the identification of paraphyly or polyphyly within certain genera of tribe Fothergilleae highlights the need for targeted genomic investigations and further evaluation of current taxonomic boundaries. Collectively, this phylogeny, grounded in comprehensive plastid genome data, provides a foundational framework for future taxonomic studies and comparative investigations of biogeography and morphological evolution in Hamamelidaceae.

## Data Availability

The datasets presented in this study can be found in online repositories. The names of the repository/repositories and accession number(s) can be found below: https://www.ncbi.nlm.nih.gov/genbank/, PX119977- PX119991.

## Supplementary material

Supplementary Figure 1 Mauve alignment of twenty-eight Hamamelidaceae plastid genomes. Local collinear blocks within each alignment are represented by blocks of the same color 3 connected with lines.

Supplementary Figure 2 Comparison of junctions of large single-copy (LSC), small single-copy (SSC), and inverted repeat regions (IRs) in the plastid genomes among 28 species in Hamamelidaceae. The “*ycf1**” gene with an asterisk mark refers to the truncated ycf1 (*ψycf1*) in IRb. JLB is junction between LSC and IRb, JSB is junction between SSC and IRb, JSA is junction between SSC and IRa and JLA is junction between LSC and IRa.

Supplementary Figure 3 Phylogenetic trees constructed based in comparative analyses. **(A)** Maximum likelihood (ML) tree constructed based on concatenated dataset of non-coding region (NCG) of Hamamelidaceae plastid genomes. **(B)** Maximum likelihood tree constructed based on concatenated dataset of protein coding regions (PCGs-concat) of Hamamelidaceae plastid genomes. **(C)** Phylogenetic tree constructed based on coalescent analysis of protein coding region (PCGs-coal) of Hamamelidaceae plastid genomes. Values along branch represent ML bootstrap percentages (only BS < 100% for **A**, **B**) and posterior probabilities (only PP < 1.00 for **C**).
