# Supplementary material for: Plastid genome evolution and phylogenomics with broad taxon sampling: insights into intrafamilial classification of Hamamelidaceae
Source: Front Plant Sci. 2026 Aug 26;17:1926463. doi: 10.3389/fpls.2026.1926463 (PMC13553752; doi:10.3389/fpls.2026.1926463)
Supplement: Supplementary file 1 [file Image2.pdf]

Inverted Repeats

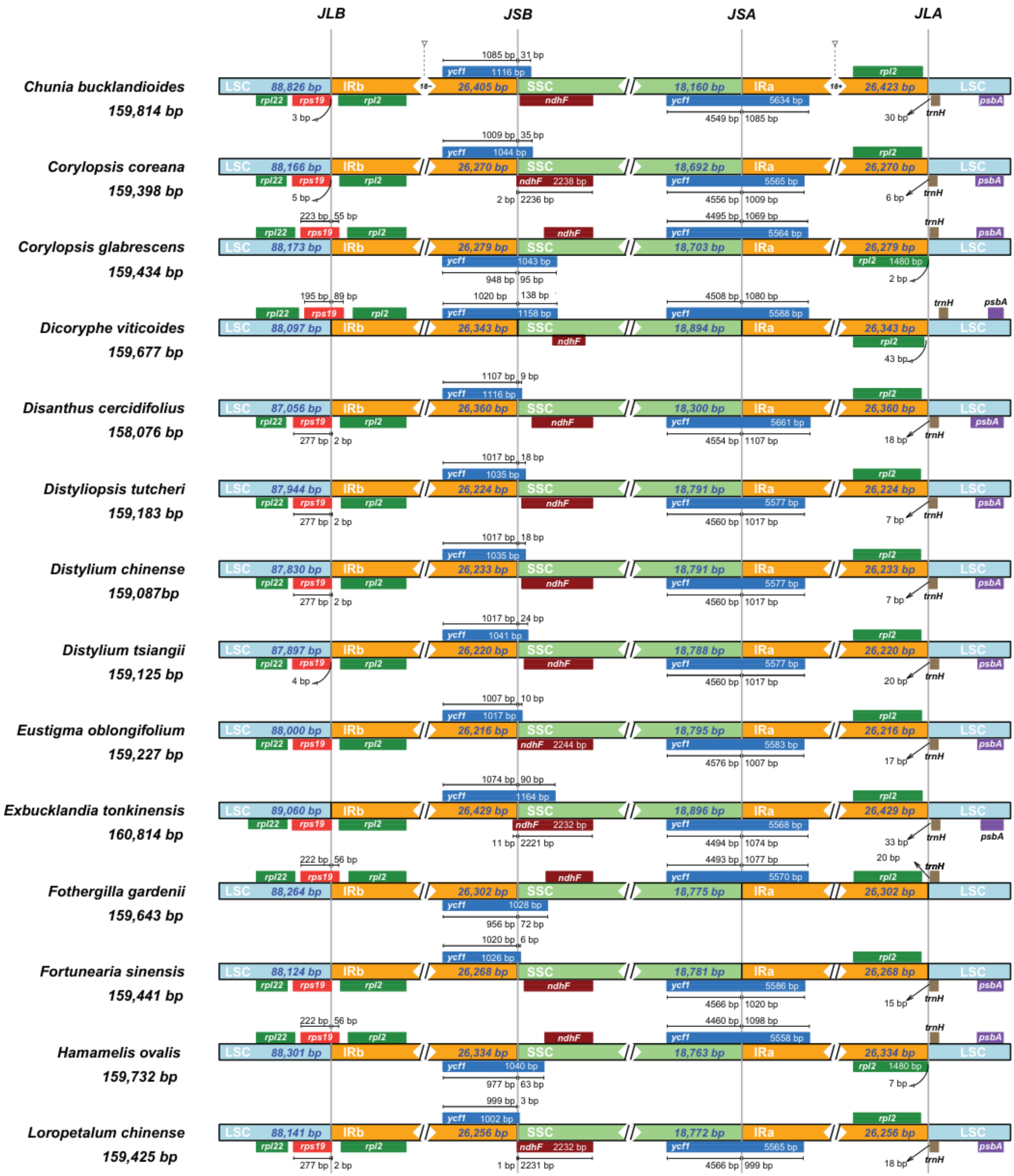

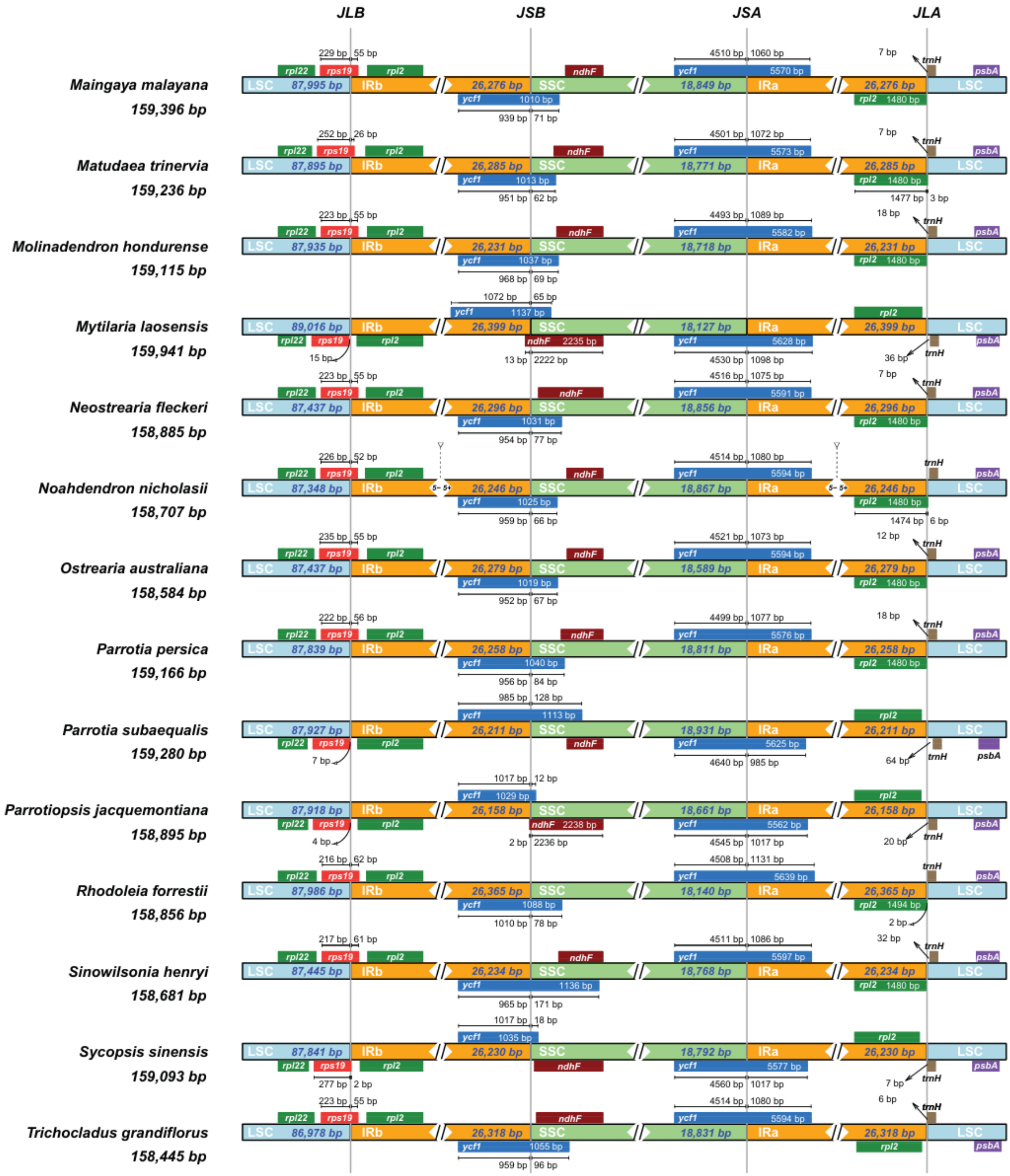

JLB
JSB
JSA
JLA
Maingaya malayana
159,396 bp
LSC 87,995 bp
IRb 26,276 bp
SSC 18,849 bp
IRa 26,276 bp
Matudaea trinervia
159,236 bp
LSC 87,895 bp
IRb 26,285 bp
SSC 18,771 bp
IRa 26,285 bp
Molinadendron hondurense
159,115 bp
LSC 87,935 bp
IRb 26,231 bp
SSC 18,718 bp
IRa 26,231 bp
Mytilaria laosensis
159,941 bp
LSC 89,016 bp
IRb 26,399 bp
SSC 18,127 bp
IRa 26,399 bp
Neostrearia fleckeri
158,885 bp
LSC 87,437 bp
IRb 26,296 bp
SSC 18,856 bp
IRa 26,296 bp
Noahdendron nicholasii
158,707 bp
LSC 87,348 bp
IRb 26,246 bp
SSC 18,867 bp
IRa 26,246 bp
Ostrearia australiana
158,584 bp
LSC 87,437 bp
IRb 26,279 bp
SSC 18,589 bp
IRa 26,279 bp
Parrotia persica
159,166 bp
LSC 87,839 bp
IRb 26,258 bp
SSC 18,811 bp
IRa 26,258 bp
Parrotia subaequalis
159,280 bp
LSC 87,927 bp
IRb 26,211 bp
SSC 18,931 bp
IRa 26,211 bp
Parrotiopsis jacquemontiana
158,895 bp
LSC 87,918 bp
IRb 26,158 bp
SSC 18,661 bp
IRa 26,158 bp
Rhodoleia forrestii
158,856 bp
LSC 87,986 bp
IRb 26,365 bp
SSC 18,140 bp
IRa 26,365 bp
Sinowilsonia henryi
158,681 bp
LSC 87,445 bp
IRb 26,234 bp
SSC 18,768 bp
IRa 26,234 bp
Sycopsis sinensis
159,093 bp
LSC 87,841 bp
IRb 26,230 bp
SSC 18,792 bp
IRa 26,230 bp
Trichocladus grandiflorus
158,445 bp
LSC 86,978 bp
IRb 26,318 bp
SSC 18,831 bp
IRa 26,318 bp
rpl22
rps19
rpl2
ndhF
ycf1
trnH
psbA
