# Supplementary material for: Plastid genome evolution and phylogenomics with broad taxon sampling: insights into intrafamilial classification of Hamamelidaceae
Source: Front Plant Sci. 2026 Aug 26;17:1926463. doi: 10.3389/fpls.2026.1926463 (PMC13553752; doi:10.3389/fpls.2026.1926463)
Supplement: Supplementary file 2 [file Image3.pdf]

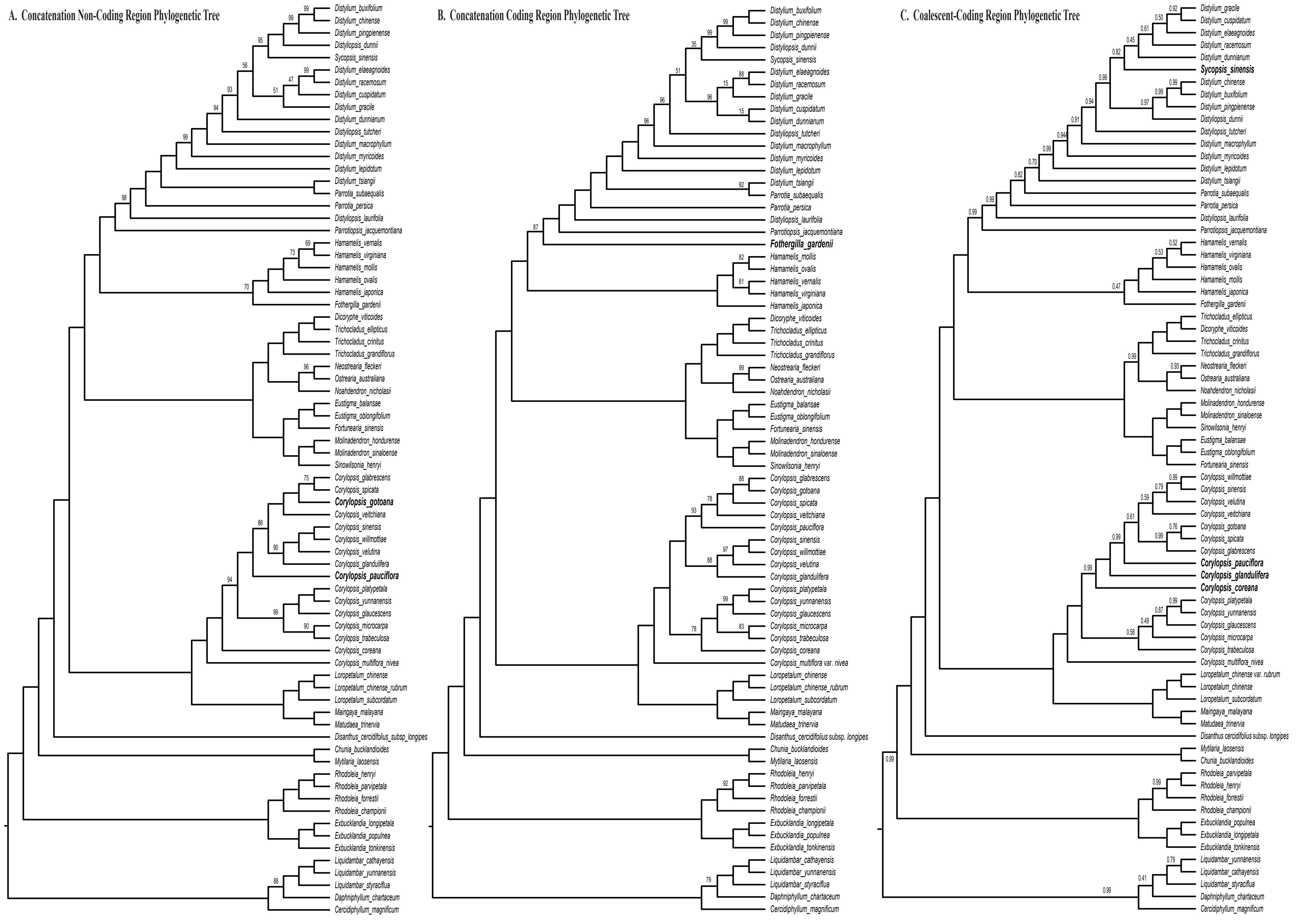
A. Concatenation Non-Coding Region Phylogenetic Tree
Distylium_buxifolium
Distylium_chinense
Distylium_pingpienense
Distyliopsis_dunnii
Sycopsis_sinensis
Distylium_elaeagnoides
Distylium_racemosum
Distylium_cuspidatum
Distylium_gracile
Distylium_dunnianum
Distyliopsis_tutcheri
Distylium_macrophyllum
Distylium_myricoides
Distylium_lepidotum
Distylium_tsiangii
Parrotia_subaequalis
Parrotia_persica
Distyliopsis_laurifolia
Parrotiopsis_jacquemontiana
Hamamelis_vernalis
Hamamelis_virginiana
Hamamelis_mollis
Hamamelis_ovalis
Hamamelis_japonica
Fothergilla_gardenii
Dicoryphe_viticoides
Trichocladus_ellipticus
Trichocladus_crinitus
Trichocladus_grandiflorus
Neostrearia_fleckeri
Ostrearia_australiana
Noahdendron_nicholasii
Eustigma_balansae
Eustigma_oblongifolium
Fortunearia_sinensis
Molinadendron_hondurense
Molinadendron_sinaloense
Sinowilsonia_henryi
Corylopsis_glabrescens
Corylopsis_spicata
Corylopsis_gotoana
Corylopsis_veitchiana
Corylopsis_sinensis
Corylopsis_willmottiae
Corylopsis_velutina
Corylopsis_glandulifera
Corylopsis_pauciflora
Corylopsis_platypetala
Corylopsis_yunnanensis
Corylopsis_glaucescens
Corylopsis_microcarpa
Corylopsis_trabeculosa
Corylopsis_coreana
Corylopsis_multiflora_nivea
Loropetalum_chinense
Loropetalum_chinense_rubrum
Loropetalum_subcordatum
Maingaya_malayana
Matudaea_trinervia
Disanthus_cercidifolius_subsp_longipes
Chunia_bucklandioides
Mytilaria_laosensis
Rhodoleia_henryi
Rhodoleia_parvipetala
Rhodoleia_forrestii
Rhodoleia_championii
Exbucklandia_longipetala
Exbucklandia_populnea
Exbucklandia_tonkinensis
Liquidambar_cathayensis
Liquidambar_yunnanensis
Liquidambar_styraciflua
Daphniphyllum_chartaceum
Cercidiphyllum_magnificum
B. Concatenation Coding Region Phylogenetic Tree
Distylium_buxifolium
Distylium_chinense
Distylium_pingpienense
Distyliopsis_dunnii
Sycopsis_sinensis
Distylium_elaeagnoides
Distylium_racemosum
Distylium_gracile
Distylium_cuspidatum
Distylium_dunnianum
Distyliopsis_tutcheri
Distylium_macrophyllum
Distylium_myricoides
Distylium_lepidotum
Distylium_tsiangii
Parrotia_subaequalis
Parrotia_persica
Distyliopsis_laurifolia
Parrotiopsis_jacquemontiana
Fothergilla_gardenii
Hamamelis_mollis
Hamamelis_ovalis
Hamamelis_vernalis
Hamamelis_virginiana
Hamamelis_japonica
Dicoryphe_viticoides
Trichocladus_ellipticus
Trichocladus_crinitus
Trichocladus_grandiflorus
Neostrearia_fleckeri
Ostrearia_australiana
Noahdendron_nicholasii
Eustigma_balansae
Eustigma_oblongifolium
Fortunearia_sinensis
Molinadendron_hondurense
Molinadendron_sinaloense
Sinowilsonia_henryi
Corylopsis_glabrescens
Corylopsis_gotoana
Corylopsis_spicata
Corylopsis_veitchiana
Corylopsis_pauciflora
Corylopsis_sinensis
Corylopsis_willmottiae
Corylopsis_velutina
Corylopsis_glandulifera
Corylopsis_platypetala
Corylopsis_yunnanensis
Corylopsis_glaucescens
Corylopsis_microcarpa
Corylopsis_trabeculosa
Corylopsis_coreana
Corylopsis_multiflora var. nivea
Loropetalum_chinense
Loropetalum_chinense_rubrum
Loropetalum_subcordatum
Maingaya_malayana
Matudaea_trinervia
Disanthus_cercidifolius subsp. longipes
Chunia_bucklandioides
Mytilaria_laosensis
Rhodoleia_henryi
Rhodoleia_parvipetala
Rhodoleia_forrestii
Rhodoleia_championii
Exbucklandia_longipetala
Exbucklandia_populnea
Exbucklandia_tonkinensis
Liquidambar_cathayensis
Liquidambar_yunnanensis
Liquidambar_styraciflua
Daphniphyllum_chartaceum
Cercidiphyllum_magnificum
C. Coalescent-Coding Region Phylogenetic Tree
Distylium_gracile
Distylium_cuspidatum
Distylium_elaeagnoides
Distylium_racemosum
Distylium_dunnianum
Sycopsis_sinensis
Distylium_chinense
Distylium_buxifolium
Distylium_pingpienense
Distyliopsis_dunnii
Distyliopsis_tutcheri
Distylium_macrophyllum
Distylium_myricoides
Distylium_lepidotum
Distylium_tsiangii
Parrotia_subaequalis
Parrotia_persica
Distyliopsis_laurifolia
Parrotiopsis_jacquemontiana
Hamamelis_vernalis
Hamamelis_virginiana
Hamamelis_ovalis
Hamamelis_mollis
Hamamelis_japonica
Fothergilla_gardenii
Trichocladus_ellipticus
Dicoryphe_viticoides
Trichocladus_crinitus
Trichocladus_grandiflorus
Neostrearia_fleckeri
Ostrearia_australiana
Noahdendron_nicholasii
Molinadendron_hondurense
Molinadendron_sinaloense
Sinowilsonia_henryi
Eustigma_balansae
Eustigma_oblongifolium
Fortunearia_sinensis
Corylopsis_willmottiae
Corylopsis_sinensis
Corylopsis_velutina
Corylopsis_veitchiana
Corylopsis_gotoana
Corylopsis_spicata
Corylopsis_glabrescens
Corylopsis_pauciflora
Corylopsis_glandulifera
Corylopsis_coreana
Corylopsis_platypetala
Corylopsis_yunnanensis
Corylopsis_glaucescens
Corylopsis_microcarpa
Corylopsis_trabeculosa
Corylopsis_multiflora_nivea
Loropetalum_chinense var. rubrum
Loropetalum_chinense
Loropetalum_subcordatum
Maingaya_malayana
Matudaea_trinervia
Disanthus cercidifolius subsp. longipes
Mytilaria_laosensis
Chunia_bucklandioides
Rhodoleia_parvipetala
Rhodoleia_henryi
Rhodoleia_forrestii
Rhodoleia_championii
Exbucklandia_populnea
Exbucklandia_longipetala
Exbucklandia_tonkinensis
Liquidambar_yunnanensis
Liquidambar_cathayensis
Liquidambar_styraciflua
Daphniphyllum_chartaceum
Cercidiphyllum_magnificum
